# The Relationship between Individual and Family Characteristics and Cyberbullying Exposure in a Nationally Representative Sample of School-Aged Children Living in Serbia

**DOI:** 10.3390/ijerph18147443

**Published:** 2021-07-12

**Authors:** Ljiljana Rakic, Milena Santric-Milicevic, Dejan Nikolic, Milena Vasic, Uros Babic, Jovana Todorovic, Zorica Terzic-Supic, Sanja Milenkovic

**Affiliations:** 1 Clinic for Hematology, University Clinical Center of Serbia, 11000 Belgrade, Serbia; lj.rakic68@gmail.com; 2 Institute of Social Medicine, Centre-School of Public Health and Health Management, Faculty of Medicine, University of Belgrade, 11000 Belgrade, Serbia; jovana.todorovic@med.bg.ac.rs (J.T.); zorica.terzic-supic@med.bg.ac.rs (Z.T.-S.); 3 Faculty of Medicine, University of Belgrade, 11000 Belgrade, Serbia; dejan.nikolic@udk.bg.ac.rs (D.N.); uros.babic@med.bg.ac.rs (U.B.); 4 Department of Physical Medicine and Rehabilitation, University Children’s Hospital, 11000 Belgrade, Serbia; 5 Institute of Public Health of Serbia, 11000 Belgrade, Serbia; milena_vasic@batut.org.rs; 6 Faculty of Dentistry in Pancevo, University Business Academy of Novi Sad, 26000 Pancevo, Serbia; 7 Clinic of Urology, University Clinical Center of Serbia, 11000 Belgrade, Serbia; 8 Institute of Hygiene and Medical Ecology, Faculty of Medicine, University of Belgrade, 11000 Belgrade, Serbia; sanja.milenkovic@med.bg.ac.rs

**Keywords:** cyberbullying, exposure, prevalence, predictors, family, Serbia

## Abstract

The study provides evidence on the individual and family factors as potential predictors (odds ratio—OR and 95% CI) of cyber-violence among school-aged children (11–17 years old) from 64 schools participating in the 2017 Serbian Study on health behavior in school-age children (HBSC). The standardized international HBSC research protocol was used. The study population was the nationally representative sample of 3267 students of V and VII grades of primary and I grade of secondary schools in Serbia. Potential predictors for the probability of occurrence vs. non-occurrence of cyberbullying exposure at least once and multiple times were identified among 24 explanatory variables, including the individual characteristics and family context. The cyberbullying exposure was more prevalent among girls than among boys of school-age, i.e., over one in seven girls and one in ten boys were exposed to cyberbullying. Over one in seven students at age 13 years and almost every seventh student at grade I of the gymnasium were exposed to cyberbullying. There were more students exposed to at least one cyberbullying than to multiple cyberbullying. Potential predictors of exposure to cyberbullying are gender, opinion of the family’s affluence status, fathers’ employment, communication with father, and family support. The study compensates for the evidence of cyberbullying in Serbia, which could help raise awareness, inform national and international stakeholders in the region and enable their efforts and strengthen cooperation in ending cyberbullying. This study’s findings could inform the development of an intervention program aimed at families and various professionals involved in protecting and improving school-age children’s health and well-being.

## 1. Introduction

The modern society is characterized by the fourth industrial revolution, the exponential evolution of “a fusion of technologies that blurs the lines between the physical, digital, and biological spheres” that offers another environment for human beings to live, work and socialize in. Recent estimates are that 53% of the global population has internet access [1]. Today’s school-age children are born and raised with technological innovations and tend to use cyberspace to connect and share information. The frequency of internet use among youth of 16–19 years old in the Europe region considerably increased over the period from 2011 to 2019, with the lowest reported in Turkey, 41% (2012), and the highest in Croatia, Latvia, Malta, United Kingdom, Iceland and North Macedonia 100% (2019) [2]. Youth more frequently use the Internet than the general population. For example, in 2019, 96% of youth vs. 83% of individuals have ever used the Internet in Serbia [2]. The use of digital technologies and artificial intelligence can support increased efficiency and productivity, intercultural understanding and cohesion, and advances in living standards. There is also the possibility of cyber-technology abuse, which forces us to redefine our moral boundaries, structures, habits, relationships, regulations, and policies. Cyberbullying among school-age children is a form of a child’s loss of control over privacy and personal data due to someone’s attempts to injure, harass, insult and deliberately attack other children through social media platforms and technological devices in school and outside [3]. Therefore, cyberbullying is considered a form of indirect aggression [4]. The difficult peculiarity of the cyberbullying phenomenon is that it can be almost endless even after a single act; for example, cyberbullies anonymously and without consent post or produce and share someone’s picture or video for the purpose of humiliation. In this form of violence among young people, the perpetrators feel the power of repeating cyberbullying, almost indefinitely, using cyberspace’s breadth and depth, which ultimately contributes to the destruction of the victim’s life. The United Nations Children’s Fund found that 33% of school-aged children from 30 countries reported cyberbullying in 2019 [5]. The average prevalence of cyberbullying exposure among school-aged children has been 12% for boys and 14% for girls [6], with variations among countries from 3% among 15-year-old boys in Spain to 29% among 15-year-old boys in Lithuania [7]. To date, many countries have also indicated that at age 13, girls are more than boys exposed to at least once cyberbullying, with the respective prevalence varying within the range 27% vs. 22% (Latvia, 2017/18) to 6% vs. 4% (Spain, 2017/18) [6,7].

Plenty of literature highly discusses the relationship between the utilization of the Internet and cyberbullying [8,9,10,11,12,13,14,15,16]. As everyday use of the Internet is common among school-age children, the quality of time they spend with their families may be relevant to reducing their predisposition to cyberbullying [17,18,19]. Arguments about the protective potential of the paternal style [18,19], monitoring [20], and the relationship between parents and children [21,22,23,24,25,26,27] against cyberbullying are ongoing. However, to our knowledge, in Serbia, none of the similar research on a representative sample has comprehensively explained cyberbullying exposure within an array of family factors.

The various theoretical and empirical approaches that emerge from the socio-ecological theory help understand cyberbullying of school-aged children. The socio-ecological theory [28] explains that violence, in this case, cyberbullying, is a product of combined influences of biological, social, cultural, and economic factors that affect the individual, interpersonal relationships, community, and the entire society [28]. For most of these factors, beyond the individual, such as the fundamental cognitive perspective, interaction models, and coping strategies are established within the family environment. Cyberbullying exposure and aggression seem to be often present among adolescents from single-parent and other types of non-nuclear families [29]. Family relationships can undoubtedly affect children’s behavior, development (including physical, psychological and cognitive, social and emotional, sexuality and gender identity), progress, and social interactions, including resilience to cyberbullying and vice versa.

The Global Strategy for Women’s, Children’s, and Adolescents’ Health (2016–2030) [30], among other international policy initiatives, calls for better monitoring and consideration of cyberbullying. Scoping the family factors of school-aged children is needed both locally and internationally for providing a comprehensive legally binding document and a proper community preparation for cyberbullying prevention and protection and victims support. In Serbia, several national legislative documents emphasize the role of family for the youth safety, health, and development, including the following: Law on Youth, Strategy for Youth Development (2015–2025), Law on Public Heath, National Program for Health Care of Women, Children and Youth (2009) and the Decree that regulates preventive measures for the safety and protection of children when using information and communication technologies (the Internet) [31,32,33,34,35]. However, despite their importance, family factors are less analyzed in contexts outside of domestic violence in the Serbian literature [36]. Researchers mainly report on children exposed to physical or psychological violence types [37,38,39,40,41,42], while predictors of cyberbullying exposure are less studied. Multidisciplinary research in Serbia shows that bullying begins in early childhood [37], has the gender dimension, and that more than two-thirds of the school-aged children were at least once exposed to internet risk [36]. Repeated cyberbullying threatens to humiliate and undermine children and may also develop cumulatively into severe cases. Knowing that in a virtual environment, a single violent act often becomes repetitive [43], we hypothesize that a criterion for prevention and protection from cyberbullying should be exposure to a single and not repetitive acts. It is essential to identify common elements for exposure to single and multiple cyberbullying acts and examine factors associated with repeated victimization [44]. Acting upon the predictors could help prevent isolated cyberbullying events and exacerbation into multiple and repetitive behaviors [44,45].

The WHO Regional Office for Europe Health information gateway [46] does not contain data on cyberbullying among school-aged children from Serbia. Since 1983, a network of 45 countries across Europe and North America study cross-nationally health behavior in school-aged children (hereinafter referred to as HBSC). The new cyberbullying issues were presented in the recent HBSC survey covering the school year 2017/2018 [6,7]. The Republic of Serbia, the European Union (EU) candidate country, has applied HBSC methodology for the first time in the spring of 2017 on a nationally representative sample of schools [42] to prove the national capacity to conduct in full the methodology and the protocol of the HBSC study. After the successful piloting, in 2018, the Republic of Serbia has been accepted for official membership in the network of countries conducting the HBSC study [6,47]. Comparing the successive HBSC findings for 2017 and 2018, progress can be detected, if any. The Serbian National Contact Center for Child Safety on the Internet [48] points out that cyberbullying needs a better understanding of the family context’s problem to prevent its escalation among school-aged children. The international [6,7] and both Serbian HBSC reports [40,47] contain some information on cyberbullying (i.e., data by gender and family affluence status). However, Serbia lacks comprehensive, nationally representative findings regarding cyberbullying’s potential predictors amongst the family characteristics collected through HBSC surveys. Effective action at a national or international level can be wisely tailored only upon comprehending cyberbullying at a local level. In this study, we try to compensate for that kind of evidence for Serbia, inform national and international stakeholders, and enable their efforts to strengthen cooperation in stopping cyberbullying among school-age children. The study objective is to examine the relationship between individual and family characteristics and cyberbullying exposure among school-aged children (11–17 years) living in Serbia in a nationally representative sample of primary and secondary schools. The research’s main purpose is to heighten the public health stakeholders, families, and school awareness about the family issues related to the school-aged children who experienced cyberbullying.

## 2. Methods

### 2.1. Study Design and Sample

This study was a secondary analysis of 2017 HBSC data focusing on cyberbullying in a nationally representative sample of 3267 students of 64 schools (V and VII grades of primary and I grade of secondary schools) in Serbia [40]. The Institute of Public Health of Serbia (IPHS), with the support of the World Health Organization (WHO), the Ministry of Health, and the Ministry of Education, Science and Technological Development conducted the HBSC survey as a pilot study with the standardized (2013/2014) international HBSC research protocol, from 3 May to 8 June 2017 on the territory of the Republic of Serbia (not including data for Kosovo and Metohija) [40].

According to the international HBSC survey methodology, the HBSC study for 2017 in Serbia was conducted on a nationally representative sample [40]. The sample was selected to provide statistically reliable indicators by region for primary and secondary schools. There were four statistical regions: Belgrade, Vojvodina, Sumadija and Western Serbia, Southern and Eastern Serbia. Schools were selected using the probability proportional to size algorithm, where the school’s size is determined by the number of students of a given grade. The primary unit sampling for each grade was the school or the class (each class of the sample should contain 1500 children), yielding the sample of 64 schools (38 schools as the main sample and additional 26 schools as substitutes for each class because of potential refusals to participate in the survey). After selecting the schools, a list of classes is nominated using a random number in each school. Replacements were activated when the main sample has not reached the expected number of participants. The sampled schools were informed on the research protocol, ethical aspects, instrument, and objectives and asked to submit their consent to participate and carry out the procedure of obtaining parental consent for children’s participation in the research. In 2017, the HBSC study in Serbia had 3267 students in the national representative sample.

### 2.2. Study Instrument and Variables

Following the WHO methodology and the standardized international research protocol, the 2017 HBSC study in Serbia also used the HBSC standardized questionnaire. The standardized HBSC questionnaire was translated from English into Serbian and then back into English [40]. The Ethics Committee of IPHS gave consent for this specific secondary analysis (Decision 1934/1, 3 March 2020). Students self-completed the questionnaire voluntarily and anonymously.

The present study of cyberbullying among school-aged children in Serbia has two outcome variables and 24 explanatory variables. The response rate ranged from 91.7 to 97.5% so that the missing responses by variables were less than 9%.

#### 2.2.1. Outcome Variables

The study’s two outcomes described exposure to cyberbullying in the past couple of months: at least once and multiple times. Specific questions investigated exposure to cyberbullying [36]: “*How often did they abuse you in the following way…?*

- Someone sent malicious instant messages, posted on the wall, sent emails or text messages, or designed a website where others made fun of me;
- Someone took photos of me that are not suitable for sharing without my permission and posted them on the Internet”.

The four original possible answers were re-coded as follows:

- “I have not been abused in this way in the past couple of months”—None;
- “Once or twice”—At least once;
- “Two or three times a month; About once a week; Several times a week;”—Multiple times;
- “I don’t know/no answer”—Missing;

The four categories of answers allowed us to explore the prevalence and predictors of cyberbullying exposure, including:

- At least once versus none (Model 1);
- Multiple times versus none (Model 2); and
- Multiple times versus at least once (Module 3).

#### 2.2.2. Explanatory Variables

This study explored two sets of explanatory variables, individual and family characteristics (five and nineteen variables, respectively). The individual variables, mainly socio-demographic characteristics, include the following: gender (boys, girls), age (11–12 years, 13–14 years, 15–17 years); school type, grade (primary, V grade; primary, VII grade; gymnasium I grade; and vocational school, I grade); life satisfaction (measured on a scale from 0 (worst) to 10 (best, and then re-coded into 1—very bad life, 2—bad life, 3—average life, 4—good life, 5—very good life), and region of their residence (Belgrade, Vojvodina, Sumadija and Western Serbia, Southern, and Eastern Serbia). The family context variables include family members, relations, communication, and support. More precisely, these variables are the following:

- The family size, i.e., number of family members (2–3 members, 4–5 members, 6–7 members, 8 and more members);
- Live with (both parents, one parent, one parent and step-father/mother, relatives/legal guardians);
- Brothers and sisters (none, one sister/brother, two or more sisters/brothers);
- Family Affluence Scale (FAS) (low, average, high);
- Father’s employment (unemployed, employed, don’t know/not seeing father);
- Mother’s employment (unemployed, employed, don’t know/not seeing mother);
- Opinion of the family financial state (bad, average, good);
- Think the important things are talked about in family (absolutely disagree, disagree, neither agree nor disagree, agree, absolutely agree);
- When a student speaks, someone in the family listens to what he/she says (absolutely disagree, disagree, neither agree nor disagree, agree, absolutely agree);
- When don’t understand something, questions are asked in student’s family;
- When there is disagreement, in student’s family, they talk until it is resolved;
- Student talks with father about the things that bother the student;
- Students talks with mother about the things that bother the student;
- Student talks with step-father about the things that bother the student;
- Students talks with step-mother about the things that bother the student;
- Family is trying really to help the student;
- Student receives from family necessary emotional help and support;
- Student can talk about his/her problems with family;
- Family is ready to help him/her in decision making.

#### 2.2.3. Family Affluence Scale (FAS)

FAS score was calculated based on the question: How many computers (computers) does your family have (including laptops and tablets, NOT including console players and smartphones)? 0—None; 1—One; 2—Two; 3—More than 2; Does your family have a car, van, or truck? 0—No, 1—Yes, one, 2—Yes, two and more; Do you have the room that you only use? 0—No, 1—Yes; Does your family have a dishwasher at home? 0—No, 1—Yes; How many bathrooms (bath/shower room or both) are in your house? 0—None; 1—One; 2—Two, 3—More than two; In the last 12 months, how many times have you traveled for holidays or holidays with your family outside Serbia? 0—None; 1—Once; 2—Twice; and 3—More than twice. The range of FAS Score was: 0–13, where respondents classified as Category I have low FAS (0–4), Category II average (5–9), and Category III have high FAS (10–13) [49].

### 2.3. Statistical Analysis

Descriptive statistics were used to present the prevalence of outcome variables in regard to explanatory variables as percentages (%) with a 95% confidence interval (CI). Analytical statistics used the Pearson chi-square test to assess the statistically significant difference (at *p* < 0.05) between categorical variables. Univariate and multivariate logistic regression (a cross-odds ratio—OR with a 95% confidence interval—CI) was calculated to quantify the strength of association between explanatory variables and four outcome variables (different bullying types and participation in fights). Based on the answers to the questions, each outcome variable had three models of predictors:

- In the first model (Model 1), the reference was “not exposed (none) (0)” versus “exposed at least once (1)”.
- In the second model (Model 2), the reference was “not exposed (none) (0)” versus “exposed multiple times (1)”.
- In the third model (Model 3), the reference was “exposed at least once (0)” versus “exposed multiple times (1)”.

All statistical analyzes were performed by the IBM SPSS Statistics for Windows, version 22.0 (IBM Corp., Armonk, NY, USA).

## 3. Results

### 3.1. The Prevalence of Cyberbullying Exposure among School-Aged Children in Serbia, 2017

In the sample of 3267 students, there were more males than females and more students at age 15–17 years (Table 1). The cyberbullying exposure was more prevalent among girls than among boys of school-age, i.e., over one in seven girls and one in ten boys were exposed to cyberbullying (Table 1). Additionally, over one in seven students at age 13 years and almost every seventh student at grade I of the gymnasium were exposed to cyberbullying.

There were more students exposed to at least one cyberbullying than to multiple cyberbullying. At least once exposure to cyberbullying (Table 1) was the most prevalent for those at the age of 13–14 years (10.3%, or over one in nine school-aged children), at VII grade of primary school (9.3%), with bad life satisfaction (17.7%), living with one brother or sister (9.3%), and with employed mother (9.3%). The prevalence of at least one exposure to cyberbullying was significantly higher among school-aged children: whose family does not talk until the disagreement is resolved (14.2%), whose family is not ready to help in decision making (15.5%), or is not trying really to help (15.7%); does not talk about important things (19.4%), does not listen to what they say (13.7%), or questions are not asked in the family when not understood (20.0%) and, among those who talk very hard with father (15.8%), or talk hard with mother (17.9%) about the things that bother them, those who neither agree nor disagree that receive from the family necessary emotional help and support (18.4%), and talk about the problems with the family (14.5%) (Table 1).

The multiple times cyberbullying exposure (Table 1) was the most prevalent among boys (3.9%), and respondents with very bad life satisfaction (14.3%), who do not know father’s (12.4%) and mother’s (10.4%) employment, with a bad opinion on the family financial state (10.8%), those who absolutely disagree that the important things are talked about (11.9%), that questions are being asked in the family if not understood (18.8%), that the family talks until resolve disagreement (11.5%), those who talk very hard with father (5.6%), or with step-mother (33.3%) about the things that bother them, those who disagrees that family is trying really to help them (8.2%), those who absolutely don’t receive necessary emotional help and support from the family (8.1%), and cannot talk about the problems with the family (8.2%) and those who absolutely disagree that their family is ready to help them in decision making (9.0%) (Table 1).

Table 2 shows that boys and girls who have been victims of cyberbullying differ in age, school grade, and family wealth status. These aspects are relevant for defining the target population for protection against cyberbullying. In the observed age groups, girls were most exposed to cyberbullying in general at the age of 13 to 14 years (*p* < 0.001), but to at least one cyberbullying at the age of 15 to 17 years (*p* < 0.001). Boys were most exposed to cyberbullying in general at ages 15 and 17 (*p* = 0.001). At the age of 13 to 14, there were more cyberbullying victims among boys than among girls (*p* = 0.027).

When there is an intervention in schools against at least one cyberbullying (Table 2), vocational schools (the 1st grade) should focus on the protection of boys because their prevalence of exposure is highest at this level of education (*p* = 0.041). Together with the highest prevalence rates among girls, the intervention in the 7th grade of primary school and the 1st grade of gymnasium should aim at protecting girls from at least one cyberbullying (*p* < 0.001) and cyberbullying in general (*p* < 0.001). In the 7th grade of primary school and the gymnasium I, girls were more often victims than boys of at least one cyberbullying (*p* < 0.001, i.e., *p* < 0.001), as well as victims of cyberbullying in general (*p* = 0.012 and *p* = 0.002, respectively).

Suppose the family is the context for intervention against at least one cyberbullying. In that case, a target population should be girls with low and average family affluence status (*p* = 0.009 and *p* = 0.015, respectively) because their prevalence of exposure was higher than that of boys (Table 2). However, boys with a high family affluence status were more often victims of multiple cyberbullying than girls (*p* = 0.039), which indicates the need for a different preventive intervention.

### 3.2. Models of Cyberbullying Exposure among School-Aged Children in Serbia, 2017

In Model 1, the individual variables such as gender (girls), age (older than 11 years), school grade (primary school grade VII and higher) were positively associated with once-twice cyberbullying exposure. Family variables that were negatively associated with once-twice cyberbullying exposure (Model 1) are as follows: opinion that family financial status is average and good; listening to each other; talk to resolve the disagreements within the family and talk about the problems with the family. Family variables positively associated with once-twice cyberbullying exposure (Model 1) are as follows: not very easy talk with the father; and not absolute receiving the necessary emotional help and support from the family (Table 3).

In Model 2, the individual variables such as gender (girls) and the average, good, or very good life satisfaction were negatively associated with multiple cyberbullying exposures. Family variables positively associated with multiple cyberbullying exposures (Model 2) are: unknown employment of a father and a mother; and hard/very hard talk to the father about the problems. Family variables negatively associated with multiple cyberbullying exposures (Model 2) are as follows: perceived average and good family financial state; talks about the important things in the family; asking questions in the family if not understood; talk until the disagreement is resolved; the family trying really to help the one; receiving from the family necessary emotional help and support; talk about the problems with the family; and the family ready to help the one in decision making (Table 3).

In Model 3, the individual variables such as gender (girls), primary school VII grade, and good or very good life satisfaction were negatively associated with multiple cyberbullying exposures. Family variables positively associated with multiple cyberbullying exposures (Model 3) are unknown employment of a father and a mother, and hard/very hard talk to the father about the problems. Family variables negatively associated with multiple cyberbullying exposure (Model 3) are as follows: perceived family financial state as good; talks about the important things in the family; asking questions in the family if not understood; talk until the disagreement is resolved; the family trying really to help the one; receiving from the family necessary emotional help and support; talk about the problems with the family; and the family ready to help the one in decision making (Table 3).

Table 4 shows five predictors of cyberbullying among the Serbian school-aged children, one individual factor—gender; and four family variables—father’s employment; talk to father about things that bother them; talk with family about problems and the perception of family financial status. Girls were by 1.63 times more likely than boys exposed to at least one cyberbullying act (Model 1). However, girls were less likely by 56% than boys exposed to multiple cyberbullying after at least one such event (Model 3). Similarly, those who can easily talk to father about the things that bother them were by 65% less likely exposed to multiple cyberbullying after one such event (Model 3). Unfortunately, those who hardly or very hardly talk to father about things that bother them were more likely than their counterparts (by 1.07 times and by 2.16 times respectively), exposed to at least one event of cyberbullying (Model 1), and by 2.4 times more likely exposed to multiple cyberbullying acts (Model 2). Those who did not know whether their father is employed had by 3.15 higher likelihood than their counterparts for multiple exposures to cyberbullying (Model 2). School-aged students who perceived the financial state as average to good were by 58–68% less likely exposed to at least one cyberbullying (Model 1) and by 72–73% less likely exposed to multiple times (Model 2). Students who can talk with family about problems were by 68–69% less likely exposed than their counterparts to at least one cyberbullying (Model 1).

## 4. Discussion

In this study, we investigated the prevalence and predictors of cyberbullying exposure among school-aged children in Serbia. Cyberbullying exposure was defined as when someone via digital media bullied respondents by sending them malicious messages, emails, text messages, photos, posting them on the wall posts and websites, or photographing respondents and posting photos online without permission. In 2017, about one in seven school-aged Serbian children at age 13 years, at gymnasium grade I, or female sex were exposed to cyberbullying.

In the Serbian representative sample, there were more victims of at least one cyberbullying than of multiple cyberbullying. Overall, cyberbullying was more prevalent among Serbian girls than among boys; over one in seven girls and one in ten boys were exposed to cyberbullying. Different gender characteristics in cultural and regulatory contextual arrangements in all countries may be responsible because girls are often cyber-victims [50]. Our finding is slightly lower than the international average prevalence for boys (10.8% vs. 12%) and girls (13.1% vs. 14%) [6]. This study also showed a lower prevalence in Serbia in 2017 than in 2018 of at least one cyberbullying exposure among girls [7] and per school type and grade [40]. This finding indicates an annual increase in the age-gender prevalence of cyberbullying per school grades in Serbia and calls for urgent measures by schools and families to stop the escalation of cyberbullying. The present study findings are similar to the 2017/2018 HBSC studies’ reported prevalence of 4% among 13-year-old boys in Spain and 5% among 15-year-old girls in Greece [7]. According to gender and family affluence status, our findings correspond to the internationally typical pattern where girls with low and boys with high affluence status are more likely cyberbullying victims than their counterparts [6,7]. The prevalence in this study is also lower than reported in the HBSC study for Serbia in 2018 and is almost twice lower than the international average for girls in low (7.2% vs. 14%) and boys in high affluence status (7% vs. 12%) [7]. Such socio-demographic profile suggests that cyberbullying prevention programs in Serbia should focus on girls in low family affluence and boys in high family affluence status.

This study showed that 16 of the 24 observed individual and family variables correlated separately with cyberbullying. Furthermore, gender, opinion of the family’s affluence status, fathers’ employment, communication with father, and family support are potential predictors for cyberbullying exposure in Serbia. Girls were more likely than boys exposed to at least one cyberbullying and will be less likely to be re-exposed afterward. Cyberbullying exposure was more likely for a school-aged child who had poor communication about problems with the father and did not know its work status. On the other hand, a child who was able to talk to his family about problems and thought that the financial situation in the family was good, was less susceptible to cyberbullying Other researchers have also highlighted the relationship between the type of parental communication (offensive and avoidant vs. open and supportive communication) and cyberbullying [19,27], suggesting the need for social-educational intervention.

Our study results re-emphasize the interactions of family context, communication, and support with cyberbullying. [17,18,19,22,24,26], and to prevent conversion to cyberbullying within covert social networks among adolescents. As in other countries, in Serbia, family affluence status perception and parenting relationships are potential predictors of cyberbullying among school-aged children [18,20]. Namely, students who perceive better financial status and life are less likely to be exposed to cyberbullying. The family is the central institution of upbringing children and a cornerstone of society, and is of paramount importance for cyberbullying prevention programs [18,29,51,52,53]. A protective effect of the family is coming from the school-aged child’s factors (such as resilience, self-control, socializing with peers) and relationship to the family (such as the support, resilience, connectedness, and supervision) [18,19,20,37,54,55]. Our study confirms that talk to families about children’s problems has a protective effect from exposure to at least one cyberbullying event. Seeking support from the family is a form of a child victim’s coping mechanism [56,57]. Some victims, however, fear that a family member may encourage further shame or manipulation if they admit that they have been cyberbullied [58]. That fear might explain the study finding why children who have difficulties talking to their father are at higher risk of at least once or multiple cyberbullying. Additionally, easy conversation with the father reduces the risk of multiple exposures after at least one exposure to cyberbullying. A balanced emotional warmth and protection, spending more time in family activities helps children open and talk about their problems [16,17,52]. To enhance efforts and effective prevention of cyberbullying, stakeholders’ promotion of healthier social relationships must consider the complexities of the individual child experience and greater attachment to parents, including appropriate and effective parental control and family behavior.

The methodological strength of the present study is the use of the standardized international questionnaire, which enables the collection of the same data in all countries from the HBSC network and enables international comparisons and learning good practices [59], but due to contextual specificities, the study findings should not be generalized to other population groups. Another study strength streams from the nationally representative sample of the 2017 HBSC survey because in some countries, evidence on cyberbullying base on convenient samples of youth [51]. Even when done in a representative sample, the findings’ generalization is not recommended beyond the studied population [3]. Our study’s sample size corresponds to the other studies; it ranged from 1020 in Greenland and 2265 in Malta to 11,136 in Spain and 12,931 in Canada [60]. Since the survey results come from a nationally representative sample of school children in Serbia, they can be compared internationally and used to program regional and local activities to stop school violence across the HBSC network in line with UNESCO’s recommendations [61]. A regression modeling approach in the present study is a step further from the Serbian HBSC reports. The findings of this study can be used to develop an intervention program aimed at families and various professionals involved in protecting and improving the health and well-being of school-age children. Because of the cross-sectional design, the causal pathways between cyberbullying and individual and family variables could not be established in this study; instead, the potential predictors of single and multiple cyberbullying events were identified. The study findings inform the need for a family microsystem that helps school-aged children have life satisfaction, healthier peer relationships, and reduce cyberbullying. A set of 24 variables in the study did not account for the influence of many other aspects of family and social context, which need to be explored in new studies. In the next round of the HBSC survey, we recommend complementing information on the magnitude of various forms of cyberbullying with the parental reports and on the opinion of the impact of the social exclusion and the introduction of online education, and the COVID-19 pandemic on family context (since many children might have lost family members). The study’s findings may help design specific skills-building interventions targeting vulnerable families with school-age children and longitudinal studies on cyberbullying to determine the cause and consequences to create safer use of cyber technology. Eliminating cyber technologies abuse will make a world of difference to school-aged children’s prosperity, particularly the ability to experience pleasure with mastering new knowledge and skills to support the unfolding developmental process.

## 5. Conclusions

The cyberbullying exposure was more prevalent among girls than among boys of school-age, i.e., over one in seven girls and one in ten boys were exposed to cyberbullying. Over one in seven students at age 13 years and almost every seventh student at grade I of the gymnasium were exposed to cyberbullying. There were more students exposed to at least one cyberbullying than to multiple cyberbullying. Potential predictors of exposure to cyberbullying are gender, opinion of the family affluence status, fathers’ employment, communication with father, and family support. The study findings may contribute to a better understanding of the students’ family environment exposed to cyberbullying. The study compensates for the evidence of cyberbullying in Serbia, which could help to raise awareness, inform national and international stakeholders in the region and enable their efforts and strengthen cooperation with parents, schools, and the entire community in ending cyberbullying.

## Figures and Tables

**Table 1:** Prevalence of cyberbullying exposure among school-aged children in Serbia, 2017.

Variables	Sample * n (%)	Cyberbullying Exposure Prevalence (%) and (95% CI)		
		At Least Once	Multiple Times	Total
***Individual characteristics***				
**1. Gender**	**3267 (100)**			
Boys	1633 (51.3)	6.9 (5.6–8.1)	3.9 (3.0–4.9)	10.8 (9.3–12.3)
Girls	1553 (48.7)	10.6 (9.1–12.2)	2.4 (1.7–3.2)	13.1 (11.4–14.7)
Pearson Chi-square *p*	0.156	<0.001	0.018	0.045
**2. Age, years**	**3186 (100)**			
mean ± standard deviation	14.2 ± 1.71			
11–12	854 (26.8)	5.3 (3.8–6.8)	2.6 (1.5–3.6)	7.8 (6.0–9.6)
13–14	931 (29.2)	10.3 (8.4–12.3)	3.2 (2.1–4.4)	13.5 (11.3–15.7)
15–17	1401 (44.0)	9.7 (8.2–11.3)	3.6 (2.6–4.5)	13.3 (11.5–15.1)
Pearson Chi-square p	<0.001	<0.001	0.430	<0.001
**3. School type, grade**	**3186 (100)**			
Primary, V grade	876 (27.5)	5.3 (3.8–6.7)	2.9 (1.8–4.0)	8.1 (6.3–9.9)
Primary, VII grade	922 (28.9)	10.6 (8.6–12.6)	2.9 (1.8–4.0)	13.6 (11.3–15.8)
Gymnasium, I grade	342 (10.7)	10.5 (7.3–13.8)	4.4 (2.2–6.6)	14.9 (11.1–18.7)
Vocational school, I grade	1046 (32.8)	9.3 (7.5–11.0)	3.3 (2.3–4.4)	12.6 (10.6–14.6)
Pearson Chi-square *p*	<0.001	<0.001	0.535	<0.001
**4. Life satisfaction**	**3149 (100)**			
Very bad	28 (0.9)	7.1 (−2.4–16.7)	14.3 (1.3–27.2)	21.4 (6.2–36.6)
Bad	62 (2.0)	17.7 (8.2–27.3)	6.5 (0.3–12.6)	24.2 (13.5–34.9)
Average	520 (16.5)	14.2 (11.2–17.2)	3.5 (1.9–5.0)	17.7 (14.4–21.0)
Good	1046 (33.2)	9.8 (8.0–11.5)	2.5 (1.5–3.4)	12.2 (10.3–14.2)
Very good	1493 (47.4)	5.4 (4.2–6.5)	3.1 (2.3–4.0)	8.5 (7.1–9.9)
Pearson Chi-square *p*	<0.001	<0.001	0.004	<0.001
**5. Region**	**3186 (100)**			
Belgrade	681 (21.4)	8.8 (6.7–10.9)	4.0 (2.5–5.4)	12.8 (10.3–15.3)
Vojvodina	732 (23.0)	8.5 (6.5–10.5)	3.6 (2.2–4.9)	12.0 (9.7–14.4)
Sumadija and Western Serbia	1078 (33.8)	7.5 (5.9–9.1)	2.5 (1.6–3.4)	10.0 (8.2–11.8)
Southern and Eastern Serbia	695 (21.8)	10.6 (8.4–12.9)	3.2 (1.9–4.5)	13.8 (11.2–16.4)
Pearson Chi-square *p*	<0.001	0.152	0.353	0.087
***Family characteristics***				
**Family size (number of members)**	**3001 (100)**			
Mean ± standard deviation	4.81 ± 1.40			
2–3 members	388 (12.9)	9.0 (6.2–11.9)	3.4 (1.6–5.1)	12.4 (9.1–15.6)
4–5 members	1768 (58.9)	9.1 (7.8–10.4)	2.7 (1.9–3.4)	11.8 (10.3–13.3)
6–7 members	748 (24.9)	8.7 (6.7–10.7)	2.5 (1.4–3.7)	11.2 (9.0–13.5)
Eight and more members	97 (3.2)	8.2 (2.8–13.7)	4.1 (0.2–8.1)	12.4 (5.8–18.9)
Pearson Chi-square *p*	<0.001	0.175	0.711	0.945
**Live with**	**3001 (100)**			
Both parents	2540 (84.6)	8.6 (7.5–9.7)	2.8 (2.2–3.4)	11.4 (10.1–12.6)
One parent	368 (12.3)	10.9 (7.7–14.0)	3.0 (1.2–4.7)	13.9 (10.3–17.4)
One parent and stepfather/mother	58 (1.9)	6.9 (0.4–13.4)	1.7 (−1.6–5.1)	8.6 (1.4–15.8)
Relatives/Legal guardians	35 (1.2)	11.4 (0.9–22.0)	0	11.4 (0.9–22.0)
Pearson Chi-square *p*	<0.001	0.450	0.728	0.485
**Have brothers and sisters**	**3046 (100)**			
None	350 (11.5)	9.1 (6.1–12.2)	2.6 (0.9–4.2)	11.7 (8.3–15.1)
One	1698 (55.7)	9.3 (7.9–10.7)	2.8 (2.0–3.5)	12.1 (10.5–13.6)
Two or more	998 (32.8)	7.4 (5.8–9.0)	3.0 (1.9–4.1)	10.4 (8.5–12.3)
Pearson Chi-square *p*	<0.001	<0.001	0.895	0.734
**Family affluence (FAS)**	**3135 (100)**			
Mean ± standard deviation	7.26 ± 2.54			
Low	475 (15.2)	9.5 (6.8–12.1)	2.5(1.1–3.9)	12.0 (9.1–14.9)
Average	2020 (64.4)	8.4 (7.2–9.6)	3.1 (2.3–3.8)	11.4 (10.0–12.8)
High	640 (20.4)	9.5 (7.3–11.8)	3.8 (2.3–5.2)	13.3 (10.7–15.9)
Pearson Chi-square *p*	<0.001	0.557	0.495	0.426
**Father’s employment**	**3125 (100)**			
Unemployed	418 (13.4)	8.9 (6.1–11.6)	3.3 (1.6–5.1)	12.2 (9.1–15.3)
Employed	2594 (83.0)	8.8 (7.7–9.9)	2.7 (2.1–3.4)	11.5 (10.3–12.8)
Don’t know	113 (3.6)	9.7 (4.3–15.2)	12.4 (6.3–18.5)	22.1 (14.5–29.8)
Pearson Chi-square *p*	<0.001	0.949	<0.001	0.003
**Mother’s employment**	**3148 (100)**			
Unemployed	985 (31.3)	7.8 (6.1–9.5)	3.1 (2.1–4.2)	11.0 (9.0–12.9)
Employed	2115 (67.2)	9.3 (8.1–10.6)	2.9 (2.2–3.7)	12.2 (10.8–13.6)
Don’t know	48 (1.5)	2.1 (−2.0–6.1)	10.4 (1.8–19.1)	12.5 (3.1–21.9)
Pearson Chi-square *p*	<0.001	0.010	0.013	0.584
**Opinion on the family financial state**	**3161 (100)**			
Bad	157 (5.0)	18.5 (12.4 –24.5)	10.8 (6.0–15.7)	29.3 (22.2–36.4)
Average	986 (31.2)	10.0 (8.2–11.9)	2.7 (1.7–3.8)	12.8 (10.7–14.9)
Good	2018 (63.8)	7.4 (6.2–8.5)	2.8 (2.1–3.5)	10.2 (8.8–11.5)
Pearson Chi-square *p*	<0.001	<0.001	<0.001	<0.001
**In my family, I think the important things are talked about**	**3132 (100)**			
Absolutely disagree	42 (1.3)	14.3 (3.7–24.9)	11.9 (2.1–21.7)	26.2 (12.9–39.5)
Disagree	62 (2.0)	19.4 (9.5–29.2)	3.2 (-1.2–7.6)	22.6 (12.2–33.0)
Neither agree nor disagree	286 (9.1)	9.4 (6.1–12.8)	5.2 (2.7–7.8)	14.7 (10.6–18.8)
Agree	952 (30.4)	9.5 (7.6–11.3)	1.8 (0.9–2.6)	11.2 (9.2–13.2)
Absolutely agree	1790 (57.2)	7.8 (6.6–9.1)	3.3 (2.5–4.1)	11.1 (9.7–12.6)
Pearson Chi-square *p*	<0.001	0.011	<0.001	<0.001
**In my family, when I speak someone listens to what I say**	**3119 (100)**			
Absolutely disagree	73 (2.3)	13.7 (5.8–21.6)	5.5 (0.3–10.7)	19.2 (10.1–28.2)
Disagree	108 (3.5)	12.0 (5.9–18.2)	4.6 (0.7–8.6)	16.7 (9.6–23.7)
Neither agree nor disagree	289 (9.3)	10.0 (6.6–13.5)	4.2 (1.9–6.5)	14.2 (10.2–18.2)
Agree	1014 (32.5)	10.5 (8.6–12.3)	2.6 (1.6–3.5)	13.0 (10.9–15.1)
Absolutely agree	1635 (52.4)	7.2 (5.9–8.4)	2.9 (2.1–3.8)	10.1 (8.6–11.6)
Pearson Chi-square *p*	<0.001	0.012	0.349	0.008
**In my family, when I don’t understand something, we ask questions**	**3119 (100)**			
Absolutely disagree	38 (1.2)	10.5 (0.8–20.3)	18.8 (4.2–27.4)	26.3 (12.3–40.3)
Disagree	65 (2.1)	20.0 (10.3–29.7)	4.6 (−0.5–9.7)	24.6 (14.1–35.1)
Neither agree nor disagree	228 (7.3)	11.4 (7.3–15.5)	6.6 (3.4–9.8)	18.0 (13.0–23.0)
Agree	996 (31.9)	8.5 (6.8–10.3)	2.5 (1.5–3.5)	11.0 (9.1–13.0)
Absolutely agree	1792 (57.5)	8.2 (6.9–9.5)	2.7 (1.9–3.4)	10.9 (9.4–12.3)
Pearson Chi-square *p*	<0.001	0.010	<0.001	<0.001
**In my family, when there is disagreement, we talk until it is resolved**	**3115 (100)**			
Absolutely disagree	78 (2.5)	12.8 (5.4–20.2)	11.5 (4.4–18.6)	24.4 (14.8–33.9)
Disagree	134 (4.3)	14.2 (8.3–20.1)	3.7 (0.5–6.9)	17.9 (11.4–24.4)
Neither agree nor disagree	289 (9.3)	11.1 (7.5–14.7)	3.8 (1.6–6.0)	14.9 (10.8–10.8)
Agree	825 (26.5)	9.6 (7.6–11.6)	2.1 (1.1–3.0)	11.6 (9.4–13.8)
Absolutely agree	1789 (57.4)	7.4 (6.2–8.6)	3.0 (2.2–3.7)	10.4 (9.0–11.8)
Pearson Chi-square *p*	<0.001	0.013	<0.001	<0.001
**Can you talk with further family members about the things that bother you?**				
**Father**	**2935 (100)**			
Very easy	1472 (47.3)	6.3 (5.1–7.6)	3.2 (2.3–4.1)	9.5 (8.0–11.0)
Easy	879 (28.2)	9.9 (7.9–11.9)	1.3 (0.5–2.0)	11.1 (9.1–13.2)
Hard	388 (12.5)	12.1 (8.9–15.4)	5.2 (3.0–7.4)	17.3 (13.5–21.0)
Very hard	196 (6.3)	15.8 (10.7–20.9)	5.6 (2.4–8.8)	21.4 (15.7–27.2)
Pearson Chi-square *p*	<0.001	<0.001	<0.001	<0.001
**Step-father**	**188 (100)**			
Very easy	74 (3.3)	8.1 (1.9–14.3)	8.1 (1.9–14.3)	16.2 (7.8–24.6)
Easy	52 (2.3)	9.6 (1.6–17.6)	5.8 (−0.6–12.1)	15.4 (5.6–25.2)
Hard	29 (1.3)	10.3 (−0.7–21.4)	13.8 (1.2–26.3)	24.1 (8.6–39.7)
Very hard	33 (1.5)	15.2 (2.9–27.4)	15.2 (2.9–27.4)	30.3 (14.6–46.0)
Pearson Chi-square *p*	<0.001	0.737	0.418	0.274
**Mother**	**3048 (100)**			
Very easy	2088 (67.3)	7.5 (6.4–8.7)	2.7 (2.0–3.4)	10.2 (8.9–11.5)
Easy	682 (22.0)	10.1 (7.9–12.4)	3.7 (2.3–5.1)	13.8 (11.2–16.4)
Hard	196 (6.3)	17.9 (12.5–23.2)	4.1 (1.3–6.9)	21.9 (16.1–27.7)
Very hard	82 (2.6)	12.2 (5.1–19.3)	6.1 (0.9–11.3)	18.3 (9.9–26.7)
Pearson Chi-square *p*	<0.001	<0.001	0.168	<0.001
**Step-mother**	**149 (100)**			
Very easy	63 (2.8)	15.9 (6.8–24.9)	3.2 (−1.2–7.5)	19.0 (9.4–28.7)
Easy	38 (1.7)	5.3 (−1.8–12.4)	5.3 (−1.8–12.4)	10.5 (0.8–20.3)
Hard	18 (0.8)	5.6 (−5.0–16.1)	33.3 (11.6–55.1)	38.9 (16.4–61.4)
Very hard	30 (1.3)	10.0 (−0.7–20.7)	20.0 (5.7–34.3)	30.0 (13.6–46.4)
Pearson Chi-square p	<0.001	0.328	<0.001	0.059
**My family is trying really to help me**	**3128 (100)**			
Absolutely disagree	177 (5.7)	9.0 (4.8–13.3)	7.9 (3.9–11.9)	16.9 (11.4–22.5)
Disagree	98 (3.1)	15.3 (8.2–22.4)	8.2 (2.7–13.6)	23.5 (15.1–31.9)
Neither agree nor disagree	91 (2.9)	9.9 (3.8–16.0)	4.4 (0.2–8.6)	14.3 (7.1–21.5)
Agree	357 (11.4)	15.7 (11.9–19.5)	2.2 (0.7–3.8)	17.9 (13.9–21.9)
Absolutely agree	2405 (76.9)	7.4 (6.4–8.4)	2.5 (1.9–3.1)	9.9 (8.7–11.1)
Pearson Chi-square p	<0.001	<0.001	<0.001	<0.001
**I receive from my family necessary emotional help and support**	**3110 (100)**			
Absolutely disagree	186 (6.0)	6.5 (2.9–10.0)	8.1 (4.2–12.0)	14.5 (9.5–19.6)
Disagree	127 (4.1)	15.0 (8.8–21.2)	6.3 (2.1–10.5)	21.3 (14.1–28.4)
Neither agree nor disagree	98 (3.2)	18.4 (10.7–26.0)	2.0 (−0.8–4.8)	20.4 (12.4–28.4)
Agree	460 (14.8)	13.3 (10.2–16.4)	2.4 (1.0–3.8)	15.7 (12.3–19.0)
Absolutely agree	2239 (72.0)	7.4 (6.3–8.5)	2.5 (1.8–3.1)	9.9 (8.6–11.1)
Pearson Chi-square *p*	<0.001	<0.001	<0.001	<0.001
**With my family I can talk about my problems**	**3112 (100)**			
Absolutely disagree	195 (6.3)	10.8 (6.4–15.1)	8.2 (4.4–12.1)	19.0 (13.5–24.5)
Disagree	172 (5.5)	11.0 (6.4–15.7)	3.5 (0.7–6.2)	14.5 (9.3–19.8)
Neither agree nor disagree	117 (3.8)	14.5 (8.1–20.9)	6.8 (2.3–11.4)	21.4 (13.9–28.8)
Agree	521 (16.7)	14.2 (11.2–17.2)	1.9 (0.7–3.1)	16.1 (13.0–19.3)
Absolutely agree	2107 (67.7)	6.9 (5.8–8.0)	2.5 (1.8–3.2)	9.4 (8.2–10.6)
Pearson Chi-square *p*	<0.001	<0.001	<0.001	<0.001
**My family is ready to help me in decision making**	**3115 (100)**			
Absolutely disagree	177 (5.7)	7.9 (3.9–11.9)	9.0 (4.8–13.3)	16.9 (11.4–22.5)
Disagree	97 (3.1)	15.5 (8.3–22.7)	4.1 (0.2–8.1)	19.6 (11.7–27.5)
Neither agree nor disagree	79 (2.5)	11.4 (4.4–18.4)	5.1 (0.2–9.9)	16.5 (8.3–24.6)
Agree	417 (13.4)	14.9 (11.5–18.3)	2.6 (1.1–4.2)	17.5 (13.9–21.2)
Absolutely agree	2345 (75.3)	7.5 (6.4–8.6)	2.5 (1.8–1.1)	10.0 (8.8–11.2)
Pearson Chi-square *p*	<0.001	<0.001	<0.001	<0.001

* Response rate range 91.7–97.5%.

**Table 2:** Selected socio-demographic characteristics of boys and girls exposed to cyberbullying, Serbia 2017.

Variables	Sample * n (%)		Cyberbullying Exposure Prevalence (%) and (95% CI)								
			At Least Once			Multiple Times			Total		
	Boys	Girls	Boys	Girls	*p*	Boys	Girls	*p*	Boys	Girls	*p*
***Individual characteristics***											
**Age, years**	**1633** **(100)**	**1553** **(100)**									
11–12	414 (25.4)	440 (28.3)	2.6 (1.5–3.6)	2.7 (1.6–3.8)	0.879	1.8 (0.9–2.6)	0.8 (0.2–1.4)	0.086	4.3 (3.0–5.7)	3.5 (2.3–4.7)	0.380
13–14	464 (28.4)	467 (30.1)	3.4 (2.3–4.6)	2.0 (1.1–2.9)	<0.001	2.0 (1.1–2.9)	1.2 (0.5–1.9)	0.141	5.5 (4.0–6.9)	8.1 (6.3–9.8)	0.027
15–17	755 (46.2)	646 (41.6)	4.1 (3.1–5.2)	5.6 (4.4–6.8)	0.079	2.1 (1.4–2.9)	1.4 (0.8–2.0)	0.154	6.3 (5.0–7.6)	7.0 (5.7–8.3)	0.448
Pearson Chi-square *p*	<0.001	<0.001	0.816	<0.001		0.145	0.435		0.001	<0.001	
**School type, grade**	**1633** **(100)**	**1553** **(100)**									
Primary, V grade	429 (26.3)	447 (28.8)	2.5 (1.5–3.5)	2.7 (1.7–3.8)	0.765	2.1 (1.1–3.0)	0.8 (0.2–1.4)	0.027	4.6 (3.2–5.9)	3.5 (2.3–4.8)	0.275
Primary, VII grade	458 (28.0)	464 (29.9)	3.6 (2.4–4.8)	7.0 (5.4–8.7)	<0.001	1.7 (0.9–2.6)	1.2 (0.5–1.9)	0.332	5.3 (3.9–6.8)	8.2 (6.5–10.0)	0.012
Gymnasium, I grade	140 (8.6)	202 (13.0)	2.3 (0.7–3.9)	8.2 (5.3–11.1)	<0.001	2.0 (0.5–3.5)	2.3 (0.7–3.9)	0.794	4.4 (2.2–6.6)	10.5 (7.3–13.8)	0.002
Vocational school, I grade	606 (37.1)	440 (28.3)	4.7 (3.4–6.0)	4.6 (3.3–5.9)	0.916	2.2 (1.3–3.1)	1.1 (0.5–1.8)	0.061	6.9 (5.3–8.4)	5.7 (4.3–7.1)	0.281
Pearson Chi-square *p*	<0.001	<0.001	0.041	<0.001		0.907	0.173		0.104	<0.001	
***Family characteristics***											
**Family affluence (FAS)**	**1629** **(100)**	**1506** **(100)**									
Low	243 (14.9)	232 (15.4)	2.9 (1.4–4.5)	6.5 (4.3–8.7)	0.009	1.9 (0.7–3.1)	0.6 (−0.1–1.3)	0.081	4.8 (2.9–6.8)	7.2 (4.8–9.5)	0.133
Average	1034 (63.5)	986 (65.5)	3.4 (2.6–4.2)	5.0 (4.0–5.9)	0.015	1.7 (1.2–2.3)	1.3 (0.8–1.8)	0.306	5.1 (4.2–6.1)	6.3 (5.2–7.3)	0.119
High	352 (21.6)	288 (19.1)	4.4 (2.8–6.0)	5.2 (3.4–6.9)	0.512	2.7 (1.4–3.9)	1.1 (0.3–1.9)	0.039	7.0 (5.1–9.0)	6.3 (4.4–8.1)	0.574
Pearson Chi-square *p*	<0.001	<0.001	0.389	0.380		0.336	0.429		0.152	0.771	

* Response rate range 96.0–96.9%.

**Table 3:** Individual and family characteristics associated with cyberbullying exposure among school-aged children in Serbia, 2017: Univariate logistic regression models, OR (95% CI).

Individual and Family Characteristics	Models of Cyberbullying Exposure, OR (95% CI)		
	Model 1: None versus at Least Once	Model 2: None versus Multiple	Model 3: at Least Once versus Multiple
***Individual characteristics***			
**Gender**			
Boys	1	1	1
Girls	1.59 (1.24–2.04) **	0.64 (0.43–0.96) *	0.40 (0.25–0.64) **
**Age years**			
11–12	1	1	1
13–14	2.09 (1.44–3.01) **	1.33 (0.76–2.33)	0.64 (0.33–1.23)
15–17	1.96 (1.38–2.77) **	1.47 (0.89–2.45)	0.75 (0.41–1.38)
**School type, grade**			
Primary, V grade	1	1	1
Primary, VII grade	2.15 (1.50–3.10) **	1.09 (0.63–1.90)	0.51 (0.27–0.97) *
Gymnasium, I grade	2.1 (1.37–3.42) **	1.66 (0.86–3.19)	0.77 (0.35–1.66)
Vocational school, I grade	1.86 (1.29–2.67) **	1.23 (0.73–2.08)	0.66 (0.36–1.24)
**Life satisfaction**			
Very bad	1	1	1
Bad	2.57 (0.53–12.6)	0.47 (0.11–2.05)	0.29 (0.05–1.67)
Average	1.90 (0.44–8.26)	0.23 (0.07–0.74) *	0.18 (0.02–1.41)
Good	1.22 (0.28–5.27)	0.16 (0.05–0.48) **	0.13 (0.02–0.73) *
Very good	0.64 (0.15–2.79)	0.19 (0.06–0.57) **	0.12 (0.02–0.72) *
***Family characteristics***			
**Family size (number of members)**			
2–3 members	1	1	1
4–5 members	1.00 (0.68–1.47)	0.79 (0.42–1.47)	0.79 (0.38–1.61)
6–7 members	0.95 (0.62–0.46)	0.75 (0.37–1.53)	0.79 (0.35–1.78)
Eight and more members	0.55 (0.21–1.45)	1.19 (0.38–3.74)	2.15 (0.50–9.28)
**Live with**			
Both parents	1	1	1
One parent	1.30 (0.91–1.86)	1.10 (0.58–2.10)	0.84 (0.41–1.73)
One parent, stepfather/mother	0.78 (0.28–2.17)	0.60 (0.08–4.39)	0.77 (0.08–6.98)
Relatives/legal guardians	1.33 (0.47–3.81)	0 (0)	0 (0)
**Have brothers and sisters**			
None	1	1	1
One	1.02 (0.69–1.52)	1.08 (0.52–2.23)	1.06 (0.47–2.37)
Two or more	0.80 (0.52–1.23)	1.15 (0.54–2.45)	1.44 (0.62–3.38)
**Family affluence (FAS)**			
Low	1	1	1
Average	0.88 (0.62–1.24)	1.21 (0.65–2.26)	1.38 (0.68–2.77)
High	1.02 (0.68–1.53)	1.51 (0.75–3.05)	1.48 (0.67–3.26)
**Father’s employment**			
Unemployed	1	1	1
Employed	0.99 (0.68–1.42)	0.81 (0.45–1.45)	0.82 (0.42–1.61)
Don’t know	1.24 (0.61–2.53)	4.17 (1.92–9.07) **	3.36 (1.24–9.15) *
**Mother’s employment**			
Unemployed	1	1	1
Employed	1.21 (0.92–1.59)	0.95 (0.61–1.47)	0.78 (0.47–1.30)
Don’t know	0.27 (0.04–1.99)	3.36 (1.25–9.10)*	12.40 (1.39–110.60) *
**Perception of family financial status**			
Bad	1	1	1
Average	0.44 (0.28–0.70) **	0.21 (0.11–0.39) **	0.64 (0.33–1.26)
Good	0.32 (0.20–0.49) **	0.20 (0.11–0.36) **	0.47 (0.22–0.97)*
**In my family, I think the important things are talked about**			
Absolutely disagree	1	1	1
Disagree	1.29 (0.44–3.80)	0.26 (0.05–1.42)	0.20 (0.03–1.35)
Neither agree nor disagree	0.57 (0.22–1.49)	0.38 (0.13–1.12)	0.67 (0.17–2.56)
Agree	055 (0.22–1.36)	0.23 (0.09–0.61) **	0.51 (0.15–1.72)
Absolutely agree	0.46 (0.19–1.11)	0.13 (0.04–0.36) **	0.23 (0.06–0.83) *
**In my family, when I speak, someone listens to what I say**			
Absolutely disagree	1	1	1
Disagree	0.85 (0.35–2.07)	0.82 (0.21–3.18)	0.96 (0.20–4.54)
Neither agree nor disagree	0.69 (0.32–1.49)	0.71 (0.22–2.29)	1.03 (0.27–3.95)
Agree	0.71 (0.35–1.43)	0.44 (0.15–1.29)	0.61 (0.18–2.11)
Absolutely agree	0.47 (0.23–0.94) *	0.48 (0.17–1.38)	1.03 (0.31–3.43)
**In my family, when I don’t understand something, we ask questions**			
Absolutely disagree	1	1	1
Disagree	1.86 (0.55–6.25)	0.29 (0.07–1.23)	0.39 (0.09–1.59)
Neither agree nor disagree	0.97 (0.32–3.00)	0.37 (0.13–1.05)	0.22 (0.06–0.80) *
Agree	0.67 (0.23–1.96)	0.13 (0.05–0.35) **	0.20 (0.05–0.75) *
Absolutely agree	0.64 (0.22–1.86)	0.14 (0.06–0.36) **	0.15 (0.03–0.91) *
**In my family, when there is disagreement, we talk until it is resolved**			
Absolutely disagree	1	1	1
Disagree	1.02 (0.44–2.33)	0.30 (0.10–0.93)	0.29 (0.08–1.11)
Neither agree nor disagree	0.77 (0.36–1.65)	0.29 (0.12–0.74) **	0.38 (0.12–1.18)
Agree	0.64 (0.32–1.30)	0.22 (0.10–0.46) **	0.44 (0.17–1.15)
Absolutely agree	0.49 (0.25–0.98) *	0.15 (0.07–0.36) **	0.24 (0.08–0.68) **
**In the family, can you talk with further family members about the things that bother you?**			
**Father**			
Very easy	1	1	1
Easy	1.60 (1.18–2.17) **	0.40 (0.21–0.77)	1.42 (0.66–3.08)
Hard	2.10 (1.45–3.04) **	1.77 (1.03–3.02) *	1.36 (1.14–1.90)
Very hard	2.88 (1.86–4.47) **	2.02 (1.03–3.99) *	1.20 (0.51–2.85)
**Step-father**			
Very easy	1	1	1
Easy	1.17 (0.34–4.09)	0.71 (0.17–2.97)	0.60 (0.10–3.72)
Hard	1.41 (0.32–6.12)	1.88 (0.48–7.29)	1.33 (0.20–8.71)
Very hard	2.25 (0.63–8.08)	2.25 (0.63–8.08)	1.00 (0.19–5.36)
**Mother**			
Very easy	1	1	1
Easy	0.56 (0.28–1.11)	1.42 (0.88–2.30)	1.02 (0.59–1.76)
Hard	0.79 (0.39–1.60)	1.75 (0.82–3.74)	0.64 (0.28–1.46)
Very hard	1.53 (0.72–3.28)	2.50 (0.97–6.44)	1.40 (0.46–4.28)
**Step-mother**			
Very easy	1	1	1
Easy	0.30 (0.06–1.46)	1.50 (0.20–11.2)	5.00 (0.42–59.7)
Hard	0.46 (0.05–4.01)	7.29 (1.36–39.1)	10.0 (1.28–78.1)
Very hard	0.73 (0.18–2.92)	13.9 (2.47–78.3)	30.0 (2.22–405.9)
**My family is trying really to help me**			
Absolutely disagree	1	1	1
Disagree	1.84 (0.86–3.92)	1.12 (0.45–2.79)	0.61 (0.20–1.87)
Neither agree nor disagree	1.06 (0.45–2.51)	0.54 (0.17–1.69)	0.51 (0.13–2.02)
Agree	1.76 (0.97–3.17)	0.29 (0.12–0.70) **	0.39 (0.18–0.84) *
Absolutely agree	0.76 (0.44–1.29)	0.29 (0.16–0.53) **	0.16 (0.06–0.46) **
**I receive from my family necessary emotional help and support**			
Absolutely disagree	1	1	1
Disagree	0.92 (0.50–1.69)	0.85 (0.35–2.07)	0.34 (0.11–1.03)
Neither agree nor disagree	2.81 (1.64–4.80) **	0.27 (0.06–1.22)	0.27 (0.12–0.60) **
Agree	2.31 (1.38–3.87) **	0.30 (0.14–0.67) **	0.14 (0.05–0.39) **
Absolutely agree	1.91 (1.40–2.61) **	0.29 (0.16–0.52) **	0.09 (0.02–0.46) **
**With my family, I can talk about my problems**			
Absolutely disagree	1	1	1
Disagree	0.97 (0.50–1.88)	0.40 (0.15–1.06)	0.41 (0.14–1.28)
Neither agree nor disagree	1.39 (0.70–2.77)	0.86 (0.35–2.08)	0.62 (0.21–1.79)
Agree	1.27 (0.76–2.14)	0.27 (0.15–0.49) **	0.48 (0.23–0.99) *
Absolutely agree	0.57 (0.35–0.93) *	0.23 (0.10–0.51) **	0.18 (0.07–0.45) **
**My family is ready to help me in decision making**			
Absolutely disagree	1	1	1
Disagree	2.02 (0.93–4.40)	0.47 (0.15–1.46)	0.39 (0.10–1.54)
Neither agree nor disagree	1.43 (0.59–3.47)	0.56 (0.18–1.73)	0.29 (0.13–0.63) **
Agree	1.89 (1.03–3.49)	0.29 (0.13–0.65) **	0.23 (0.06–0.87) *
Absolutely agree	0.88 (0.50–1.55)	0.25 (0.14–0.45) **	0.16 (0.06–0.41) **

1 = reference; * *p* < 0.05; ** *p* <0.01.

**Table 4:** Potential predictors of cyberbullying exposure OR (95% CI) among school-aged children in Serbia, 2017: Multivariate logistic regression models.

Individual and Family Characteristics	Models of Cyberbullying Exposure, OR (95% CI)		
	Model 1: None versus at Least Once	Model 2: None versus Multiple	Model 3: at Least Once versus Multiple
***Individual characteristics***			
**Gender**			
Boys	1	1	1
Girls	1.63 (1.26–2.10) **	0.72 (0.47–1.10)	0.44 (0.26–0.75) **
**Age, years**			
11–12	1	\	\
13–14	0.98 (0.13–7.54)	\	\
15–17	2.53 (0.22–29.10)	\	\
**School type, grade**			
Primary, V grade	1	\	1
Primary, VII grade	2.05 (0.27–15.60)	\	0.57 (0.28–1.19)
Gymnasium, I grade	0.76 (0.07–8.78)	\	1.18 (0.49–2.86)
Vocational school, I grade	0.66 (0.06–7.53)	\	0.63 (0.30–1.33)
**Life satisfaction**			
Very bad	\	1	1
Bad	\	0.41 (0.07–2.20)	0.16 (0.01–2.41)
Average	\	0.39 (0.11–1.43)	0.13 (0.01–1.53)
Good	\	0.31 (0.09–1.11)	0.15 (0.01–1.77)
Very good	\	0.37 (0.11–1.30)	0.33 (0.03–3.88)
***Family characteristics***			
**Father’s employment**			
Unemployed	\	1	1
Employed	\	1.11 (0.58–2.13)	0.82 (0.37–1.82)
Don’t know	\	3.15 (1.25–7.92) *	2.91 (0.78–10.8)
**Mother’s employment**			
Unemployed	\	1	1
Employed	\	0.92 (0.58–1.45)	0.67 (0.38–1.20)
Don’t know	\	1.14 (0.34–3.82)	2.42 (0.19–30.50)
**Opinion on the family financial state**			
Bad	1	1	1
Average	0.42 (0.26–0.67) **	0.28 (0.15–0.59) **	0.82 (0.33–2.04)
Good	0.32 (0.21–0.51) **	0.27 (0.14–0.54) **	0.72 (0.29–1.78)
**In my family, I think the important things are talked about**			
Absolutely disagree	\	1	1
Disagree	\	0 (0)	0 (0)
Neither agree nor disagree	\	0.84 (0.17–4.21)	3.98 (0.25–63.4)
Agree	\	0.61 (0.12–3.00)	1.33 (0.09–18.9)
Absolutely agree	\	1.10 (0.23–5.29)	1.78 (0.13–24.9)
**In my family, when I speak, someone listens to what I say**			
Absolutely disagree	1	\	\
Disagree	0.82 (0.30–2.26)	\	\
Neither agree nor disagree	0.66 (0.27–1.64)	\	\
Agree	0.80 (0.34–1.87)	\	\
Absolutely agree	0.77 (0.33–1.80)	\	\
**In my family, when I don’t understand something, we ask questions**			
Absolutely disagree	\	1	1
Disagree	\	0.75 (0.10–5.80)	0.13 (0.00–3.91)
Neither agree nor disagree	\	0.84 (0.17–4.20)	0.81 (0.03–25.1)
Agree	\	0.42 (0.09–2.02)	0.29 (0.01–9.10)
Absolutely agree	\	0.41 (0.08–2.00)	0.17 (0.01–5.59)
**In my family, when there is disagreement, we talk until it is resolved**			
Absolutely disagree	1	1	1
Disagree	1.06 (0.41–2,71)	0.36 (0.07–1.70)	0.09 (0.01–1.63)
Neither agree nor disagree	0.79 (0.32–1.95)	0.56 (0.15–2.10)	0.73 (0.05–11.2)
Agree	0.89 (0.37–2.12)	0.45 (0.12–1.66)	0.72 (0.04–12.0)
Absolutely agree	1.00 (0.42–2.39)	0.53 (0.14–1.96)	0.79 (0.05–13.5)
**In the family, can you talk with further family members about the things that bother you?**			
**Father**			
Very easy	1	1	1
Easy	1.36 (0.98–1.89)	0.52 (0.25–1.09)	0.35 (0.15–0.80) *
Hard	1.07 (1.03–2.48) *	2.26 (0.95–5.34)	1.07 (0.47–2.44)
Very hard	2.16 (1.28–3.63) **	2.40 (1.2–4.71) *	0.70 (0.21–2.27)
**My family is trying really to help me**			
Absolutely disagree	\	1	1
Disagree	\	1.85 (0.36–9.51)	4.65 (0.36–60.0)
Neither agree nor disagree	\	0.90 (0.13–6.51)	0.83 (0.05–15.2)
Agree	\	0.66 (0.10–4.58)	0.61 (0.05–7.41)
Absolutely agree	\	0.85 (0.14–5.11)	1.20 (0.11–13.3)
**I receive from my family necessary emotional help and support**			
Absolutely disagree	1	1	1
Disagree	0.30 (0.12–0.81)	0.88 (0.20–3.90)	0.05 (0–1.00)
Neither agree nor disagree	1.56 (0.77–3.13)	0.55 (0.07–4.24)	0.07 (0–2.32)
Agree	1.58 (0.79–3.10)	0.89 (0.16–5.09)	0.10 (0–2.69)
Absolutely agree	1.22 (0.83–1.83)	1.07 (0.19–5.87)	0.13 (0–3.49)
**With my family, I can talk about my problems**			
Absolutely disagree	1	1	1
Disagree	0.50 (0.20–1.27)	0.69 (0.15–3.24)	3.58 (0.24–53.2)
Neither agree nor disagree	0.61 (0.26–1.41)	2.33 (0.43–12.5)	4.53 (0.29–69.7)
Agree	0.32 (0.14–0.76) *	0.93 (0.18–4.81)	1.92 (0.12–32.0)
Absolutely agree	0.31 (0.13–0.74) **	0.94 (0.20–4.56)	3.68 (0.21–63.0)
**My family is ready to help me in decision making**			
Absolutely disagree	\	1	1
Disagree	\	0.55 (0.11–2.83)	1.41 (0.09–21.9)
Neither agree nor disagree	\	0.32 (0.04–2.35)	1.38 (0.07–28.4)
Agree	\	0.44 (0.08–2.35)	0.41 (0.03–6.59)
Absolutely agree	\	0.44 (0.10–2.03)	0.57 (0.04–9.26)

1 = reference; * *p* < 0.05; ** *p* < 0.01.

## Data Availability

All data are presented in this study. Original data are available on request from IPHS.
